# Case report: A promising neoadjuvant treatment option for individuals with locally advanced HER2-positive breast cancer involves the use of Pyrotinib Maleate in combination with Trastuzumab and Pertuzumab

**DOI:** 10.1016/j.heliyon.2024.e34511

**Published:** 2024-07-11

**Authors:** Chi Pan, Lan Ge, Huifeng Zhang, Kai Sang, Jian Zhou, Tongbo Yi, Qingtao Ni

**Affiliations:** a Department of General Surgery, The Affiliated Taizhou People’s Hospital of Nanjing Medical University, Taizhou School of Clinical Medicine, Nanjing Medical University, Taizhou, Jiangsu, PR China; b Department of Pathology, The Affiliated Taizhou People’s Hospital of Nanjing Medical University, Taizhou School of Clinical Medicine, Nanjing Medical University, Taizhou, Jiangsu, PR China; c Department of Oncology, The Affiliated Taizhou People’s Hospital of Nanjing Medical University, Taizhou School of Clinical Medicine, Nanjing Medical University, Taizhou, Jiangsu, PR China

**Keywords:** Case report, Pyrotinib maleate, Trastuzumab, Pertuzumab, Her2-positive

## Abstract

Breast cancer (BC) is the prevailing malignancy among women, with HER2 overexpression observed in 20–30 % of all BC, thereby serving as a prognostic indicator for unfavorable outcomes in affected individuals. There is a necessity to establish innovative treatment protocols to expand the therapeutic alternatives accessible for managing HER2-positive BC. In this study, we report a case of HER2-positive BC that was managed in our department using a combination of three targeted drugs (Trastuzumab, Pertuzumab and Pyrotinib) along with chemotherapy. The treatment resulted in a pathological complete response (pCR) and was observed to be well-tolerated, without any significant adverse reactions. Hence, the combination of Pyrotinib and Dual HER2 blockade treatment shows promise as a neoadjuvant therapy for locally advanced HER2-positive BC to achieve a pCR in surgery. Nevertheless, this conclusion necessitates additional validation via meticulously designed clinical research investigations encompassing larger patient populations.

Key Points: Pyrotinib Maleate combined with Trastuzumab and Pertuzumab is a potential target therapy for advanced HER2-positive breast cancer patients.

## 1 Introduction

Breast cancer (BC) is the most prevalent form of cancer among women. Projections for 2023 indicate that the incidence of BC in the United States will reach 297,790 cases [1]. In China, BC has the highest incidence and death rate globally, accounting for 18.4 % of all BC cases and 17.1 % of all BC-related deaths worldwide in 2020 [2]. The survival of BC patients is influenced by clinical factors such as tumor stage, tumor grade, estrogen receptor (ER) and progesterone receptor (PR) status, as well as human epidermal growth factor receptor 2 (HER2) status [3]. HER2 overexpression is observed in 20–30 % of BC, serving as an indicator of an unfavorable prognosis among BC patients [4]. Nevertheless, the advent of precision medicine has led to substantial enhancements in the prognosis of patients with HER2-positive BC through the utilization of HER2-targeted therapies such as Trastuzumab (Herceptin®) [5]. Without Herceptin therapy, patients with HER-2 positive BC patients had a more adverse prognosis [6]. Compared with Trastuzumab monotherapy, the combination of Trastuzumab and Pertuzumab alongside chemotherapy has demonstrated a significant enhancement overall survival [7]. Pyrotinib is a new oral, irreversible pan-ErbB tyrosine kinase inhibitor (TKI) with potent anti-HER1, anti-HER2 and anti-HER4 effects [8]. There is a necessity to establish innovative treatment protocols to expand the therapeutic alternatives accessible for managing HER2-positive BC. This case study presents a patient with locally advanced HER2-positive BC who underwent treatment with a combination of three targeted drugs (Trastuzumab, Pertuzumab and Pyrotinib) in conjunction with chemotherapy, resulting in the achievement of a pathological complete response (*pCR*) in our department.

## 2 Case report

**Fig. 1:**
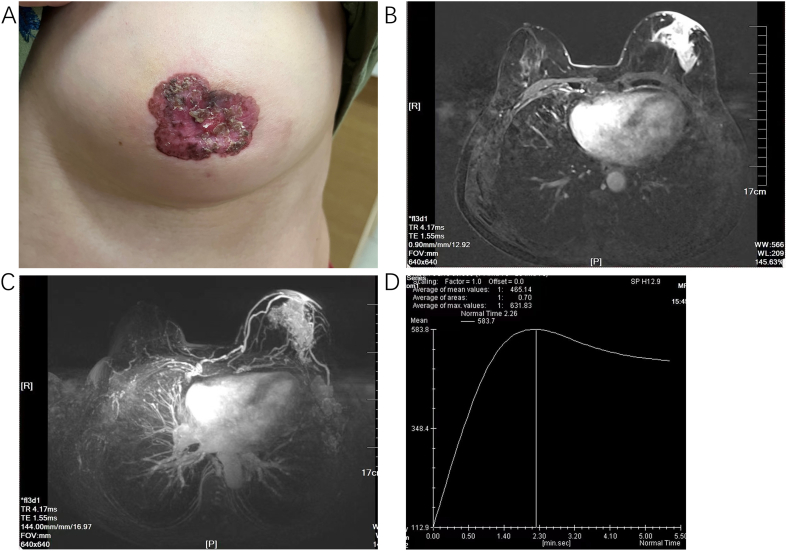
**Appearance of breasts and magnetic resonance images from the patient at initial diagnosis**. (A) Appearance of breasts of this patient. (B) T2 fat saturation imaging revealed high signal at the rear of the left nipple, about 51 × 60 × 36 mm in size. (C) Enhanced magnetic resonance imaging. (D) The signal-intensity time curves of tumors.

**Fig. 2:**
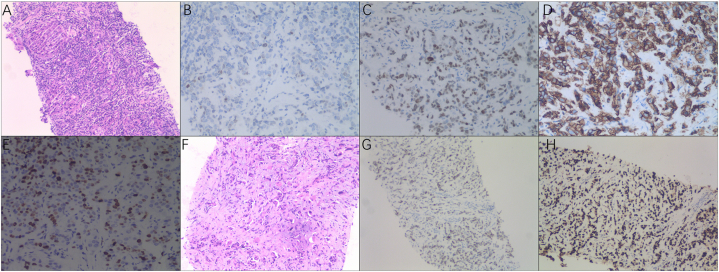
**Pathology images and immunostaining images of the patient**. (A) Pathology images of core needle biopsy of breast lumps in June 2022 (H&E staining, × 100). (B) ER immunostaining of breast lumps ( × 200). (C) PR immunostaining staining of breast lumps ( × 200). (D) HER2 immunostaining of breast lumps ( × 200). (E) Ki67 immunostaining of breast lumps ( × 200). **(F)** Pathological images of core needle biopsy of the axillary mass in June 2022 (HE staining, × 100). (G) PR immunostaining of the axillary mass ( × 100). (H) P53 immunostaining of the axillary mass ( × 100). H&E, hematoxylin and eosin; ER, estrogen receptor; PR, progesterone-receptor; HER2, Human epidermal growth factor receptor-2.

A 64-year-old woman was referred to our hospital with pruritus and ulceration of the left nipple in June 25, 2022. Physical examination showed rash with ulceration centered at the nipple and a palpable mass that was non-painful upon palpation and did not exhibit any nipple discharge. A palpable mass of 5.5 × 5.0 cm was detected posterior to the left nipple (Fig. 1A). T2 fat saturation imaging revealed a prominent signal with a heterogeneous density, measuring approximately 51 mm × 60 mm × 36 mm in size (Fig. 1B C and D). Laboratory examinations revealed high carcinoembryonic antigen (CEA) level (14.66 ng/ml). Subsequently, a core needle biopsy was performed on June 28, 2022, resulting in the pathological diagnosis of invasive ductal carcinoma. The biopsy results showed that the patient was positive for estrogen receptor (ER) (+, 2 %), progesterone receptor (PR) (++, 30 %) and P53 (+++). In addition, HER-2 was overexpressed (+++) in this case, with a low proliferation index Ki-67 (20 %) (Fig. 2A–H). The patient exhibited good health and had no medical history of hypertension or diabetes, nor did she engage in smoking or alcohol consumption. There was no family history of BC in her family. She had menarche at 14 years and her last menstrual cycle at the age of 51. Prior to initiating any treatment, a comprehensive examination was conducted to exclude the presence of distant metastases. The clinical stage was determined to be cT4N2M0, according to the 8th edition of American Joint Committee on Cancer staging manual [9].

**Fig. 3:**
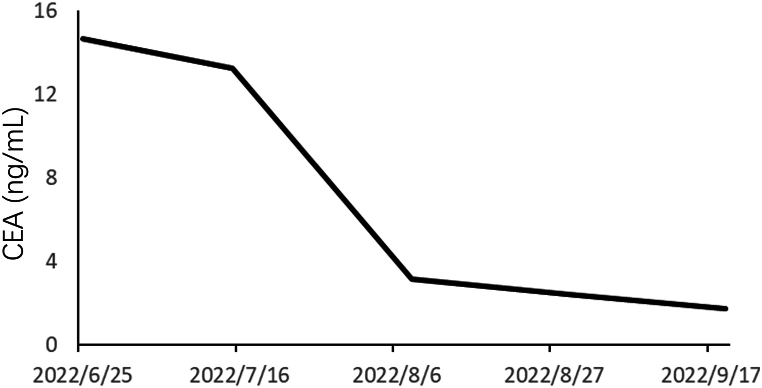
**Tumor markers after treatment with Pyrotinib combined with THP**. CEA decreased after treatment with Pyrotinib combined with THP. CEA, carcinoembryonic antigen; THP, docetaxel + trastuzumab + Pertuzumab.

**Fig. 4:**
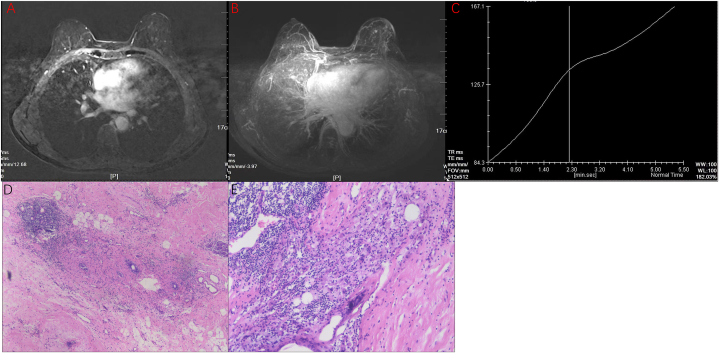
**Magnetic resonance images of the patient before surgery**. (A) T2 fat saturation imaging revealed reduced mass. (B) Enhanced magnetic resonance imaging. (C) The signal-intensity time curves of tumors. (D) Pathology images of modified radical mastectomy in June 2022. H&E staining, × 100. (E) H&E staining, × 200. H&E, hematoxylin and eosin.

**Fig. 5:**
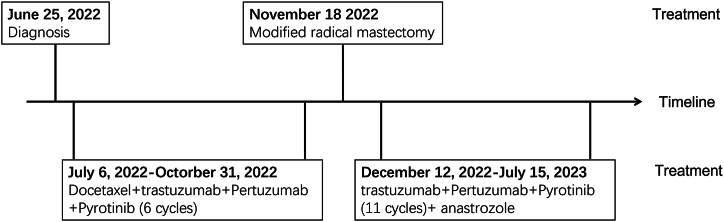
A Timeline demonstrating the treatment course of the patient.

In accordance with the Chinese Society of Clinical Oncology guidelines for BC version 2022, THP (docetaxel, 75 mg/m^2^ d1; trastuzumab, 8 mg/kg for the first cycle and 6 mg/kg thereafter d1, 21 days/cycle and Pertuzumab, 840 mg for the first cycle and 420 mg thereafter d1, 21 days/cycle) was adopted as first-line treatment for this patient. It is worth noting that in clinical trials, only 45.8 % of patients treated with THP achieved pCR [10]. A phase II study indicate that the combination of pyrotinib, trastuzumab, and chemotherapy offers a viable and safe treatment option for patients with heavily pre-treated HER2-positive metastatic BC [11]. Therefore, the administration of Pyrotinib Maleate Tablets (400mg *per os* qd) was concurrently used for better effect of tumor downstaging. Following the completion of four cycles (July 6, 2022–September 20, 2022), a gradual decline in the tumor indicator CEA was observed, ultimately returning to normal levels (Fig. 3). A reexamination using mammary magnetic resonance imaging (MRI) (Fig. 4A B and C) showed a reduction in the size of the mass. Due to the reassessment findings of this patient, she underwent subsequent treatment involving two additional cycles of THP + Pyrotinib. Modified radical mastectomy was subsequently performed on November 18, 2022. The subsequent postoperative pathology revealed fibrous tissue proliferation, inflammatory cell infiltration, multinucleated giant cell and foamy histiocytes reactions (Fig. 4D and E). No residual carcinoid or ipsilateral axillary lymph node metastasis (0/8) was observed. This patient reached pCR. The patient has continued to receive triple-targeted treatment post-operatively for one year of total therapy. As a result of the patient's hormone receptor positivity, anastrozole was administered postoperatively. Fig. 5 shows the treatment course of the patient.

Throughout the treatment, the patient experienced diarrhea, granulocytopenia and hypokalemia. However, these symptoms were effectively managed with supportive treatment, leading to the patient’s recovery without any significant adverse reactions. Overall, this patient tolerated the treatment well.

## 3 Discussion

With its high incidence worldwide, BC should receive more attention from clinicians. Historically, HER2 positive status was associated with a poor prognosis for BC [12]. However, the advent of HER2-targeted therapies has led to a remarkable improvement in the prognosis for patients with HER2-positive BC [13]. Here, we report the case of a patient with locally advanced HER2-positive BC.

As a recombinant monoclonal antibody that targets HER2, Trastuzumab (Herceptin®) has demonstrated significant efficacy in reducing recurrence and mortality rates among HER2-positive early BC patients [14]. Pertuzumab is a humanized monoclonal antibody, which acts in complementary fashion with trastuzumab by binding to different domains of the tumor [15]. Chinese Society of Clinical Oncology guidelines for BC version 2022 have highlighted that, for patients with HER2-positive status, the recommended approach for new adjuvant treatment consists of either anthracycline plus paclitaxel or non-anthracycline plus trastuzumab ± pertuzumab as the first-line treatment. The addition of pertuzumab is expected to improve the rate of complete pCR, particularly in patients with HR negative and lymph node positive conditions, thereby offering additional benefits. The combination of pertuzumab trastuzumab and chemotherapy has demonstrated enhanceddisease-free survival rates in HER2-positive patients [16].

Modified radical mastectomy is widely regarded as the standard of care for the majority of patients with locally advanced BC [17]. In order to further improve the prognosis of BC, preoperative systemic therapy is recommended for local advanced BC [18]. The utilization of neoadjuvant therapy has important benefits for the patient, including downgrading inoperable BC to operable, improving rates of breast and axillary preservation, and providing drug sensitivity information to guide future treatment strategies to improve prognosis. For HER2-positive BC, neoadjuvant chemotherapy is recommended to achieve tumor downstaging and increase the likelihood of breast-conserving surgery. The implementation of d ual HER2 blockade has demonstrated the ability to augment the proportion of patients achieving pCR [19]. Therefore, THP (Docetaxel + Trastuzumab + Pertuzumab) was adopted as first-line treatment for this patient.

Pyrotinib, an irreversible pan-ErbB inhibitor, combined with capecitabine can be considered as an alternative treatment option for patients with HER2-positive metastatic BC after trastuzumab and chemotherapy [20]. In the PHEDRA study, neoadjuvant pyrotinib, trastuzumab and docetaxel significantly improved the total pCR rate compared to a placebo, trastuzumab and docetaxel regimen (41 % vs. 22 %) [21]. Therefore, “Chemotherapy + Herceptin + Pyrotinib” regimen has been included in the 2022 Committee of Breast Cancer Society guidelines for neoadjuvant HER2-positive BC. Only the 45.8 % response rate of patients treated with THP who achieved pCR was observed in clinical trials [10]. A phase II research found that pyrotinib in combination with trastuzumab and chemotherapy provides an active alternative with a favorable safety profile in patients with HER2-positive metastatic BC who have been intensively pre-treated [11]. To enhance the probability of achieving pathological complete response (pCR) in this patient, Pyrotinib was included as an adjunct to the chemotherapy regimen recommended by the Chinese Society of Clinical Oncology guidelines for Breast Cancer version 2022. Our report further demonstrates that the combination of Pyrotinib with THP exhibits a more favorable therapeutic profile. In the real world, treatment with pyrotinib shows promising effectiveness in both the initial and follow-up stages, while also maintaining an acceptable level of tolerability [22]. Promising results have been observed in the utilization of pyrotinib-based therapy for patients afflicted with HER2-positive BC metastasized to the brain [23].

A comprehensive table (Supplementary Table 1) has been compiled to summarize the recent clinical trials incorporating Pertuzumab, Trastuzumab, and Pyrotinib in BC, which can be found on the website https://classic.clinicaltrials.gov/ct2/home.To our knowledge, this is the first case report on using three HER2-targeted drugs combined with docetaxel for the treatment of a locally advanced HER2-positive BC patient, resulting in the attainment of pCR. The CTNeoBC study confirmed that BC patients who achieve pCR with neoadjuvant therapy have a better prognosis [24]. Therefore, pCR is a valid prognostic predictor for BC patients.

This patient experienced diarrhea, which is a common side effect of pertuzumab and Pyrotinib [11,17]. However, this syndrome was not serious and improved after treatment.

We report, for the first time, a case of a patient with locally advanced HER2-positive BC who was treated with three HER2-targeted drugs combined with chemotherapy, ultimately leading to the achievement of pCR following surgical intervention. The initial treatment goal was to rapidly shrink the tumor to facilitate follow-up surgery, achieve pCR and improve the overall survival rate of this patient. The clinical outcome in the current case was excellent, indicating that chemotherapy plus triple target regimen can be used as an effective regimen for locally advanced HER2-positive patients. Our case report suggests that Pyrotinib combined with dual HER2 blockade treatment has a better treatment outcome for this specific patient population. However, further studies with a larger number of HER2-positive patients are required to confirm the efficacy and response to this regimen.

## 4 Conclusion

Pyrotinib combined with dual HER2 blockade treatment is a promising neoadjuvant therapy for locally advanced HER2-positive BC to achieve pCR in surgery. However, this conclusion needs to be further verified through well-designed clinical research studies with larger patient populations.

## Consent

Written informed consent has been provided by the patient to have the case details and any accompanying images published.

## Funding

National Natural Youth Incubation Program, Taizhou School of Clinical Medicine, 10.13039/501100007289Nanjing Medical University (no. TZKY20220105).

## Ethics approval

Institutional Review Board approval was not required to publish the details of the case included in this report.

## Data availability statement

The data is not deposited in a publicly available repository. Data included in article.

## CRediT authorship contribution statement

**Chi Pan:** Writing – review & editing, Funding acquisition, Conceptualization. **Lan Ge:** Project administration, Data curation. **Huifeng Zhang:** Visualization. **Kai Sang:** Data curation. **Jian Zhou:** Data curation. **Tongbo Yi:** Project administration, Conceptualization. **Qingtao Ni:** Writing – original draft, Visualization, Investigation.

## Declaration of competing interest

The authors declare that they have no known competing financial interests or personal relationships that could have appeared to influence the work reported in this paper.

## References

[1] Siegel R.L., Miller K.D., Wagle N.S., Jemal A. (2023). Cancer statistics, 2023. CA A Cancer J. Clin..

[2] Lei S., Zheng R., Zhang S., Wang S., Chen R., Sun K., Zeng H., Zhou J., Wei W. (2021). Global patterns of breast cancer incidence and mortality: a population-based cancer registry data analysis from 2000 to 2020. Cancer Commun..

[3] Miller K.D., Nogueira L., Devasia T., Mariotto A.B., Yabroff K.R., Jemal A., Kramer J., Siegel R.L. (2022). Cancer treatment and survivorship statistics, 2022. CA A Cancer J. Clin..

[4] Park S., Nguyen M.Q., Ta H., Nguyen M.T., Lee G., Kim C.J., Jang Y.J., Choe H. (2021). Soluble cytoplasmic expression and purification of immunotoxin HER2(scFv)-PE24B as a maltose binding protein fusion. Int. J. Mol. Sci..

[5] Xu L., Liu S., Yang T., Shen Y., Zhang Y., Huang L., Zhang L., Ding S., Song F., Cheng W. (2019). Dnazyme catalyzed tyramide depositing reaction for in situ imaging of protein status on the cell surface. Theranostics.

[6] Subramaniyan V., Fuloria S., Gupta G., Kumar D.H., Sekar M., Sathasivam K.V., Sudhakar K., Alharbi K.S., Al-Malki W.H., Afzal O., Kazmi I., Al-Abbasi F.A., Altamimi A., Fuloria N.K. (2022). A review on epidermal growth factor receptor's role in breast and non-small cell lung cancer. Chem. Biol. Interact..

[7] Swain S.M., Miles D., Kim S.B., Im Y.H., Im S.A., Semiglazov V., Ciruelos E., Schneeweiss A., Loi S., Monturus E., Clark E., Knott A., Restuccia E., Benyunes M.C., Cortes J. (2020). Pertuzumab, trastuzumab, and docetaxel for HER2-positive metastatic breast cancer (CLEOPATRA): end-of-study results from a double-blind, randomised, placebo-controlled, phase 3 study. Lancet Oncol..

[8] Li Y., Qiu Y., Li H., Luo T., Li W., Wang H., Shao B., Wang B., Ge R. (2021). Pyrotinib combined with vinorelbine in HER2-positive metastatic breast cancer: a multicenter retrospective study. Front. Oncol..

[9] Cserni G., Chmielik E., Cserni B., Tot T. (2018). The new TNM-based staging of breast cancer. Virchows Arch..

[10] Kunst N., Wang S.Y., Hood A., Mougalian S.S., Digiovanna M.P., Adelson K., Pusztai L. (2020). Cost-effectiveness of neoadjuvant-adjuvant treatment strategies for women with ERBB2 (HER2)-Positive breast cancer. JAMA Netw. Open.

[11] Xie X.F., Zhang Q.Y., Huang J.Y., Chen L.P., Lan X.F., Bai X., Song L., Xiong S.L., Guo S.J., Du C.W. (2023). Pyrotinib combined with trastuzumab and chemotherapy for the treatment of human epidermal growth factor receptor 2-positive metastatic breast cancer: a single-arm exploratory phase II trial, Breast. Cancer Treat Res..

[12] Koh J., Nam S.K., Lee Y.W., Kim J.W., Lee K.W., Ock C.Y., Oh D.Y., Ahn S.H., Kim H.H., Kang K.W., Kim W.H., Lee H.Y., Lee H.S. (2019). Trastuzumab specific epitope evaluation as a predictive and prognostic biomarker in gastric cancer patients. Biomolecules.

[13] Aapro M., Cardoso F., Curigliano G., Eniu A., Gligorov J., Harbeck N., Mueller A., Pagani O., Paluch-Shimon S., Senkus E., Thurlimann B., Zaman K. (2022). Current challenges and unmet needs in treating patients with human epidermal growth factor receptor 2-positive advanced breast cancer. Breast.

[14] Cameron D., Piccart-Gebhart M.J., Gelber R.D., Procter M., Goldhirsch A., de Azambuja E., Castro G.J., Untch M., Smith I., Gianni L., Baselga J., Al-Sakaff N., Lauer S., Mcfadden E., Leyland-Jones B., Bell R., Dowsett M., Jackisch C. (2017). 11 years' follow-up of trastuzumab after adjuvant chemotherapy in HER2-positive early breast cancer: final analysis of the HERceptin Adjuvant (HERA) trial. Lancet.

[15] Scheuer W., Friess T., Burtscher H., Bossenmaier B., Endl J., Hasmann M. (2009). Strongly enhanced antitumor activity of trastuzumab and pertuzumab combination treatment on HER2-positive human xenograft tumor models. Cancer Res..

[16] von Minckwitz G., Procter M., de Azambuja E., Zardavas D., Benyunes M., Viale G., Suter T., Arahmani A., Rouchet N., Clark E., Knott A., Lang I., Levy C., Yardley D.A., Bines J., Gelber R.D., Piccart M., Baselga J. (2017). Adjuvant pertuzumab and trastuzumab in early HER2-positive breast cancer. N. Engl. J. Med..

[17] Brackstone M., Fletcher G.G., Dayes I.S., Madarnas Y., Sengupta S.K., Verma S. (2015). Locoregional therapy of locally advanced breast cancer: a clinical practice guideline. Curr. Oncol..

[18] Aebi S., Karlsson P., Wapnir I.L. (2022). Locally advanced breast cancer. Breast.

[19] Takada M., Toi M. (2020). Neoadjuvant treatment for HER2-positive breast cancer. Chin. Clin. Oncol..

[20] Xu B., Yan M., Ma F., Hu X., Feng J., Ouyang Q., Tong Z., Li H., Zhang Q., Sun T., Wang X., Yin Y., Cheng Y., Li W., Gu Y., Chen Q., Liu J., Cheng J., Geng C., Qin S., Wang S., Lu J., Shen K., Liu Q., Wang X., Wang H., Luo T., Yang J., Wu Y., Yu Z., Zhu X., Chen C., Zou J. (2021). Pyrotinib plus capecitabine versus lapatinib plus capecitabine for the treatment of HER2-positive metastatic breast cancer (PHOEBE): a multicentre, open-label, randomised, controlled, phase 3 trial. Lancet Oncol..

[21] Wu J., Jiang Z., Liu Z., Yang B., Yang H., Tang J., Wang K., Liu Y., Wang H., Fu P., Zhang S., Liu Q., Wang S., Huang J., Wang C., Wang S., Wang Y., Zhen L., Zhu X., Wu F., Lin X., Zou J. (2022). Neoadjuvant pyrotinib, trastuzumab, and docetaxel for HER2-positive breast cancer (PHEDRA): a double-blind, randomized phase 3 trial. BMC Med..

[22] Li Y., Tong Z., Wu X., Ouyang Q., Cai L., Li W., Yu Z., Han Z., Wang X., Li M., Wang H., Li L., Yang J., Niu Z., Wang Q., Xu B. (2023). Real-world treatment patterns and outcomes of pyrotinib-based therapy in patients with HER2-positive advanced breast cancer (PRETTY): a nationwide, prospective, observational study. Int. J. Cancer.

[23] Liang X., Gui X., Yan Y., Di L., Liu X., Li H., Song G. (2024). Long-term outcome analysis of pyrotinib in patients with HER2-positive metastatic breast cancer and brain metastasis: a real-world study. Oncol..

[24] Cortazar P., Zhang L., Untch M., Mehta K., Costantino J.P., Wolmark N., Bonnefoi H., Cameron D., Gianni L., Valagussa P., Swain S.M., Prowell T., Loibl S., Wickerham D.L., Bogaerts J., Baselga J., Perou C., Blumenthal G., Blohmer J., Mamounas E.P., Bergh J., Semiglazov V., Justice R., Eidtmann H., Paik S., Piccart M., Sridhara R., Fasching P.A., Slaets L., Tang S., Gerber B., Geyer C.J., Pazdur R., Ditsch N., Rastogi P., Eiermann W., von Minckwitz G. (2014). Pathological complete response and long-term clinical benefit in breast cancer: the CTNeoBC pooled analysis. Lancet.

