# Supplementary material for: Case report: A promising neoadjuvant treatment option for individuals with locally advanced HER2-positive breast cancer involves the use of Pyrotinib Maleate in combination with Trastuzumab and Pertuzumab
Source: Heliyon. 2024 Jul 11;10(14):e34511. doi: 10.1016/j.heliyon.2024.e34511 (PMC11298905; doi:10.1016/j.heliyon.2024.e34511)
Supplement: Multimedia component 1 [file mmc1.docx]

Supplementary Table 1: Summary of recent clinical trials incorporating Pertuzumab, Trastuzumab, and Pyrotinib in BC

| Status | Study Title | Conditions | Interventions | Phase | Outcome Measures |
| --- | --- | --- | --- | --- | --- |
| Not yet recruiting | Trastuzumab Combined With Pertuzumab for Adjuvant Treatment of Breast Cancer After Neoadjuvant Therapy | Breast Cancer | Drug: Pyrotinib  Drug: Trastuzumab | Phase 3 | Invasive Disease-Free Survival (IDFS)  Disease-Free Survival (DFS)  overall survival (OS)  Distant Disease-Free Survival (DDFS) |
| Not yet recruiting | Pyrotinib Versus Pertuzumab in Combination With Neoadjuvant Trastuzumab and Nab-Paclitaxel in HER2+ Early or Locally Advanced Breast Cancer | HER2+ Early or Locally Advanced Breast Cancer | Drug: pyrotinib + trastuzumab + nab-paclitaxel  Drug: pertuzumab + trastuzumab + nab-paclitaxel | Phase 2 | tpCR (totally pathological Complete Response) assessed by the IRC(Independent Review Committee)  iDFS (Invasive disease-free survival)  EFS (event free survival) |
| Active, not recruiting | Pyrotinib Plus Trastuzumab Versus Pertuzumab Plus Trastuzumab | Breast Cancer | Drug: Pyrotinib Plus Trastuzumab  Drug: Pertuzumab Plus Trastuzumab |  | PFS  Adverse Events |
| Recruiting | A Study of ARX788 Combined With Pyrotinib Maleate Versus TCBHP (Trastuzumab Plus Pertuzumab With Docetaxel and Carboplatin) as Neoadjuvant Treatment in HER2-positive Breast Cancer Patients | HER2-positive Breast Cancer | Drug: ARX788  Drug: Pyrotinib maleate  Drug: Trastuzumab | Phase 2 Phase 3 | Total pathological complete response rate (tpCR) in percentage  Total breast pathological complete remission rate (bpCR) in percentage  Residual tumor burden (RCB) classification in grades |
| Active, not recruiting | Pyrotinib, Trastuzumab, Pertuzumab and Nab-paclitaxel as Neoadjuvant Therapy in HER2-positive Breast Cancer | Breast Cancer Invasive | Drug: Pyrotinib  Drug: Trastuzumab  Drug: Pertuzumab | Phase 2 | Percentage of Participants With Total Pathologic Complete Response (tpCR)  Percentage of Participants With Breast Pathologic Complete Response (bpCR)  Clinical response |
| Not yet recruiting | Capecitabine Plus Pyrotinib Versus Capecitabine Plus Trastuzumab and Pertuzumab in the First-line Treatment of HER2-positive Metastatic Breast Cancer | Metastatic Breast Cancer | Drug: Capecitabine and Pyrotinib  Drug: Capecitabine, Trastuzumab, and Pertuzumab | Phase 2 | Progression Free Survival  Adverse Events and Serious Adverse Events  Overall Survival |
| Recruiting | Pyrotinib Combined With Trastuzumab, Dalpiciclib, Letrozole Versus TCbHP (Trastuzumab Plus Pertuzumab With Docetaxel and Carboplatin) as Neoadjuvant Treatment in HR + / HER2 + Breast Cancer | Breast Cancer | Drug: Pyrotinib  Drug: Trastuzumab  Drug: Dalpiciclib | Phase 2 | Pathologic Complete Response Rate (tpCR: ypT0-is/ypN0)  Best overall response  Breast Pathologic Complete Response Rate (bpCR: ypT0-is)  Residual cancer burden (RCB) |
| Not yet recruiting | Pyrotinib in Breast Cancer Patients With Poor Response to the Neoadjuvant Treatment of Trastuzumab and Pertuzumab | Breast Cancer | Drug: Pyrotinib | Phase 2 | tpCR  Objective response rate (ORR)  Disease free survival (DFS) |
| Not yet recruiting | [Clinical Study of Pyrotinib in Neoadjuvant Therapy of HR-positive and HER2-positive Breast Cancer](https://classic.clinicaltrials.gov/ct2/show/NCT05430347?term=Pertuzumab%2C+Trastuzumab%2C+Pyrotinib&cond=Breast+Cancer&draw=2&rank=9) | Breast Cancer | Combination Product: Neoadjuvant therapy: TCbHPy  Combination Product: Neoadjuvant therapy: TCbHP | Phase 2 | tpCR rate (ypT0/is ypN0)  iDFS  EFS |
| Recruiting | Compare Continuation of Original Targeted Therapy With Trastuzumab Combined With Pyrotinib and Capecitabine as Postoperative Adjuvant Therapy in Non-pCR Patients With HER2 Positive Early Breast Cancer | Breast Cancer  Adjuvant Therapy | Drug: Pyrotinib+ Trastuzumab+ Capecitabine  Drug: Trastuzumab+Pertuzumab/ Trastuzumab | Phase 2 | iDFS |
| Recruiting | Pyrotinib as Neoadjuvant Agent for Non-objective Response HER2-positive Early Breast Cancer | Breast Cancer | Drug: Pyrotinib  Drug: Trastuzumab  Drug: Pertuzumab | Phase 2 | Pathological complete response (pCR) rate  Number of patients with grade >3 adverse events as a measure of safety and tolerability  Objective response rate (ORR) |
| Recruiting | Efficacy and Safety of Pyrotinib Maleate Combined With ARX788 Neoadjuvant Treatment in Breast Cancer Patients | Breast Neoplasms | Drug: Next-generation Site-specific human epidermal growth factor receptor 2 (HER2)-targeting Antibody-drug Conjugate (ARX788) | Phase 2 | Residual tumor burden (RCB) classification in grades  Best overall response rate (BORR) in percentage  Total pathological complete response rate (tpCR) in percentage |
| Not yet recruiting | Exploratory Study of Neoadjuvant Treatment of HER2-positive Breast Cancer With Py in Combination With HP | Breast Cancer | Drug: Pyrotinib Maleate Tablets | Phase 4 | pCR rate  RFS  OS |
| Recruiting | Pyrotinib in Women With High-risk in Early Stage Breast Cancer | HER2-positive Breast Cancer | Drug: Pyrotinib | Phase 2 | Invasive Disease-free Survival (iDFS) at year 2  Disease-free Survival at year 2 (2y-DFS)  Overall Survival (OS) |
| Not yet recruiting | TKIs vs. Pertuzumab in HER2+ Breast Cancer Patients With Active Brain Metastases (HER2BRAIN) | HER2-positive Breast Cancer  Brain Metastases | Drug: Trastuzumab  Drug: Taxanes  Drug: Pertuzumab  Drug: Tyrosine kinase inhibitor | Phase 2 | Objective Response Rate (ORR)  Objective Response Rate 2 (ORR2)  Progression-free Survival (PFS) |
| Not yet recruiting | Pyrotinib Plus Trastuzumab After First-line TH (P) Treatment With HER-2 Positive Breast Cancer | Advanced Breast Cancer | Drug: pyrotinib | Phase 2 | Incidence of first progression with brain metastases  ORR without CNS  TTBM |
| Recruiting | Adjuvant Study of Pyrotinib in HER-2 Positive Breast Cancer | Locally Advanced Breast Cancer | Drug: pyrotinib | Phase 3 | Invasive Disease-free Survival (iDFS)  Disease-free Survival (DFS)  Overall Survival (OS) |
| Not yet recruiting | A Study to Evaluate Inetetamab + Pyrotinib + Chemotherapy in Previously Untreated HER2-Positive Metastatic Breast Cancer | HER2-positive Recurrent/ Metastatic Breast Cancer | Drug: Inetetamab, pyrotinib, chemotherapy | Phase 2 | Progression-Free Survival (PFS)  Overall response rate (ORR)  Clinical Benefit Rate (CBR)  Number of Adverse Events using NCI CTCAE 5.0 [Safety and Tolerability] |
| Recruiting | Dynamic Observational Study With PET of 68Ga-HER2-affibody in Anti-HER2 Treatment | Breast Cancer | Drug: Docetaxel combined with Trastuzumab ± Pertuzumab  Drug: T-DM1 or Capecitabine combined with Pyrotinib regimen. |  | The correlation between the change of HER2-PET at baseline and after 2 courses of treatment and ORR.  The correlation between the change of HER2-PET at baseline and after 2 courses of treatment and PFS  The correlation between baseline HER2 expression and ORR, PFS |
| Recruiting | A Study Evaluating the Efficacy and Safety of Multiple Treatment Combinations in Participants With Breast Cancer | Inoperable, Locally Advanced or Metastatic, ER-positive Breast Cancer | Drug: Giredestrant  Drug: Abemaciclib  Drug: Ipatasertib | Phase 1 Phase 2 | Percentage of Participants with Objective Response, Defined as a Complete or Partial Response, as Determined by the Investigator According to Response Evaluation Criteria in Solid Tumors, version 1.1 (RECIST v1.1)  Number of Participants with Adverse Events, Severity Determined According to National Cancer Institute Common Terminology Criteria for Adverse Events, version 5.0 (NCI CTCAE v5.0)  Plasma Concentration of Giredestrant at Specified Timepoints |
